# The Impact of Clinical and Biochemical Frailty on Outcomes following Transcatheter Aortic Valve Replacement

**DOI:** 10.1016/j.jacadv.2026.103416

**Published:** 2026-11-03

**Authors:** Anant D. Butala, Joseph Hogarty, Shane Nanayakkara, Jason Bloom, Sonny Palmer, Samer Noaman, David McGaw, David Blusztein, Rozanne Johnston, Ellen Gardner, Elisha Gartner, Jillian Pedetti, Nay M. Htun, David M. Kaye, Antony S. Walton, Dion Stub

**Affiliations:** a Department of Cardiology, Alfred Hospital, Melbourne, Victoria, Australia; b Department of Cardiology, Cabrini Hospital, Melbourne, Victoria, Australia; c Monash-Alfred-Baker Centre for Cardiovascular Research, Monash University, Melbourne, Victoria, Australia; d Department of Cardiology, Epworth Hospital, Melbourne, Victoria, Australia; e Department of Medicine, University of Melbourne, Melbourne, Victoria, Australia; f School of Public Health and Preventive Medicine, Monash University, Melbourne, Victoria, Australia

**Keywords:** albumin, frailty, outcomes, TAVR

## Abstract

**Background:**

Frailty results from declining reserve and function and is a modifiable cardiovascular risk-factor. Despite this, there is limited evidence surrounding its role in lower-risk TAVR cohorts.

**Objectives:**

This study aimed to evaluate frailty among contemporary TAVR patients, delineate the impact of biochemical and clinical frailty, and understand its additive value to existing risk-prediction tools.

**Methods:**

Analysis of a multicenter Australian registry. Biochemical frailty (BF) was defined as serum albumin <35 g/L and clinical frailty (CF) as a Rockwood Clinical Frailty Scale ≥5. Patients meeting BF or CF criteria were categorized as frail.

**Results:**

Of the 2,661 patients, the median age 82 (IQR: 77-86), 60% male, and median STS 3.3 (2-5.2), 920 patients (35%) were considered frail. A total of 171 (19%) of frail patients fulfilled both BF and CF criteria. The frail group was older (83 vs 81, *P* < 0.001), more females (43% vs 38%; *P* = 0.016) with higher STS scores (4 vs 3, *P* < 0.001). Kaplan-Meier analysis showed a stepwise increase in 12-month mortality with increasing frailty (no frailty 4% vs BF or CF 7% vs BF + CF 16%, Log-rank *P* < 0.001). In Cox proportional hazards regression BF or CF (adjusted HR: 1.6; 95% CI: 1.0-2.4; *P* = 0.033) and BF + CF (adjusted HR: 3.3; 95% CI: 1.9-5.6; *P* < 0.001) independently predicted 12-month mortality. Addition of frailty to STS Score improved mortality prediction (area under the receiver-operating characteristic curve: 0.608 vs 0.664; DeLong’s *P* = 0.021), with Shapley analysis demonstrating significant contribution from frailty.

**Conclusions:**

Frailty is common with significant implications in TAVR. Patients with biochemical and clinical frailty criteria are highest risk. Development of strategies to address frailty is critical.

Frailty is a multidimensional syndrome characterized by increased vulnerability resulting from aging-associated decline in reserve and function across physiologic systems.^1^ It has emerged as a powerful predictor of outcomes in cardiovascular medicine, including acute coronary syndrome,^2^ heart failure,^3^ and cardiogenic shock.^4^ Importantly frailty is now considered modifiable, with preprocedural structured exercise training, nutritional counseling, psychological review, and polypharmacy optimization potentially improving outcomes and reducing mortality.^5^, ^6^, ^7^, ^8^ Given transcatheter aortic valve replacement (TAVR) patients are typically older than 70 and often comorbid, better characterization of the prevalence, risk-factors, and correlates of frailty in this population is relevant. Identifying a phenotype most at risk of experiencing the negative sequelae of frailty, and thus most likely to benefit from prehabilitation, can optimize resource utilization and improve TAVR outcomes. Furthermore, understanding the additive benefit of frailty to pre-existing risk assessment tools has the potential to improve risk-prediction, assist in periprocedural decision-making, and increase the focus on patient-centred decision-making.

Although several studies have demonstrated an association between frailty and poorer TAVR outcomes,^9^ there are important limitations in the current literature. Firstly, many prior studies evaluating frailty have focused on intermediate- to high-risk TAVR cohorts.^10^, ^11^ As the procedure has become safe and cost-effective across the spectrum of risk profiles, understanding if frailty remains prevalent and influential in younger, lower-risk, and less comorbid patients is important. Secondly, clinical and biochemical frailty have typically been considered in isolation in TAVR research. Assessing whether combining these metrics augments frailty’s impact on TAVR outcomes may allow for improved risk stratification. Thirdly, there is a paucity of research which examines frailty in Australian TAVR patients. Given Australia’s distinct aged care framework and strong transition towards home-based interventions,^12^ there is a need for region-specific analysis. Therefore, our study aimed to address these knowledge gaps, exploring frailty in TAVR using a large, multicenter Australian cohort.

## Methods

### Patient population

The Alfred-Cabrini-Epworth (ACE) TAVI Registry included patients undergoing TAVR from 2008 to 2025 for severe, symptomatic aortic stenosis (AS) across three high-volume Australian tertiary centers. The registry prospectively collected demographic, procedural, and outcomes data using REDCap (Research Electronic Data Capture) electronic data capture tool.^13^, ^14^, ^15^ This is a secure web-based software platform hosted at the Alfred Hospital. ACE Registry data are submitted to the National Australasian Cardiac Outcomes Registry TAVI database. This database is governed by the Cardiac Society of Australia and New Zealand and undergoes regular audit. Femoral, subclavian, apical, or direct aortic access was used, determined by preprocedural computed tomography, angiography, and transthoracic and/or transesophageal echocardiography. AS severity was defined as an aortic valve area <1 cm^2^ or aortic valve mean pressure gradient >40 mm Hg on transthoracic echocardiogram. When the mean pressure gradient was <40 mm Hg, AS severity was confirmed through additional testing such as dobutamine stress echocardiography or aortic valve calcium scoring. Symptoms were graded using the NYHA functional classification and all patients were reviewed by a multidisciplinary heart team before procedure.

### Consent

All participants were provided a written opt out consent for inclusion into the ACE registry before procedure. The study complied with the Declaration of Helsinki and was approved by the Alfred Hospital ethics committee (Project No: 334/26).

### Definitions and study endpoints

Clinical frailty (CF) was assessed using the Rockwood Clinical Frailty Scale (CFS).^16^ This is a widely validated measure based on clinical judgment integrating comorbidities, functional ability, and cognitive status with a scale ranging from 1 to 9 where higher scores indicate a greater degree of frailty. CFS score was derived for each patient during their preprocedural review with the structural heart team. Consistent with previous literature, CF was defined as a CFS ≥5.^16^ Biochemical frailty (BF) was defined as a serum albumin <35 g/L on preprocedural bloods. This is a widely accepted threshold for hypoproteinemia and is strongly associated with frailty.^17^ Patients meeting either BF or CF criteria were included within the “frail” group, whereas all other patients were included within the “nonfrail” group. Outcome measures were defined using the Valve Academic Research Consortium-3 criteria.^18^ The study’s primary endpoint was 12-month all-cause mortality.

### Statistical methods

Categorical variables were compared using the Pearson chi-square test or Fisher's exact test when expected cell counts were <5, with results expressed as frequencies (percentages). Continuous variables were analyzed using the Mann-Whitney *U* test and are expressed as median (IQR). Unadjusted mortality rates between the frail and nonfrail groups were compared using Kaplan-Meier curves and the log-rank test. Cox proportional hazards regression modeling was used to estimate HRs with 95% CIs for all-cause mortality at 12 months. Variables considered clinically relevant to 12-month mortality following TAVR were prespecified based on established clinical factors. They included: frailty, age, male, Society of Thoracic Surgeons (STS) score, public hospital and prior history of coronary artery disease, atrial fibrillation, stroke, chronic kidney disease, diabetes, peripheral vascular disease, anemia, left ventricular ejection fraction <40%, and valve-in-valve procedure. These variables were considered as candidate covariates and underwent univariable analysis. Among the candidate variables, those demonstrating an association with 12-month mortality at *P* < 0.10 on univariable analysis were subsequently included in the multivariable Cox proportional hazards model. Multicollinearity was assessed using variance inflation factors and adjusted generalized variance inflation factors for categorical variables. No evidence of clinically important multicollinearity was identified. Adjusted mortality curves were generated from the multivariable Cox model. Continuous covariates were fixed at their mean values, and categorical covariates were set to their reference category. The proportional hazards assumption was assessed for all covariates using Schoenfeld residuals. Missing data were handled with complete case analysis. The proportion of missing observations among variables included within the Cox model was <0.5% (Supplemental Table 1).

The predictive performance of the STS score alone and in combination with frailty for predicting 12-month mortality was evaluated using the area under the receiver-operating characteristic curve (AUC). Differences in AUC between models were compared using DeLong test. To quantify the relative contribution of each predictor to model discrimination, a Shapley value decomposition was performed. Shapley values represent the proportion of the model's incremental predictive contribution attributable to each predictor, calculated across all possible orders in which predictors can be added to the model. Higher Shapley values indicate a greater relative contribution to the model's predictive performance. Incremental predictive performance of frailty was further assessed using a censoring adjusted continuous net reclassification improvement (NRI) evaluated at 12 months. The threshold for statistical significance was a 2-tailed *P* value <0.05. R (version 4.5.1, R Foundation for Statistical Computing) was used for statistical analysis.

## Results

A total of 2,661 patients undergoing TAVR between 2008 to 2025 were analyzed; 60% were males, with a median age of 82 (IQR: 77-86) years and median STS score of 3.3 (2.0-5.2). A total of 920 patients (35%) were considered frail, with 171 (19%) fulfilling both BF and CF criteria, and the remaining 81% a single criterion (Central Illustration).

### Baseline and procedural characteristics

Baseline and procedural characteristics are compared in Table 1. The frail group was older (83 years vs 81 years; *P* < 0.001), more commonly female (43% vs 38%; *P* = 0.016) with higher STS scores (4 vs 3; *P* < 0.001). Frail patients were also more likely to reside in a care facility (6% vs 2%; *P* < 0.001) and had a higher prevalence of cardiovascular comorbidities including insulin-dependent diabetes (9% vs 6%; *P* = 0.001), peripheral vascular disease (17% vs 12%; *P* = 0.001), and chronic kidney disease (47% vs 38%; *P* < 0.001). Hemoglobin was notably lower in this cohort (121 g/L vs 130 g/L; *P* < 0.001), with almost twice the rate of anemia (45% vs 24%; *P* < 0.001). The frail group was also more symptomatic from their AS (NYHA functional class III-IV 51% vs 32%; *P* < 0.001) and had poorer left ventricular function (<40%, 10% vs 6%; *P* < 0.001). Procedural characteristics including rates of nonfemoral access (2% vs 1%; *P* = 0.576), self-expanding valves (58% vs 58%, *P* = 0.937), and valve-in-valve procedures (7% vs 5%; *P* = 0.053) were similar among frail and nonfrail patients.

**Table 1:** Baseline, Echocardiographic, and Procedural Characteristics Stratified by Frailty Status

	Frail (n = 920)	Not Frail (n = 1,741)	*P* Value
Age (y)	83 (78-87)	81 (77-86)	<0.001
Age <70 y	48 (5.2%)	96 (5.5%)	0.817
Age >90 y	81 (8.8%)	88 (5.1%)	<0.001
Male	523 (56.8%)	1,075 (61.7%)	0.016
STS score	3.97 (2.6-6)	3 (2-4.8)	<0.001
Body mass index (kg/m^2^)	27.04 (23.7-31.6)	27.46 (24.6-31.2)	0.040
Albumin (g/L)	34 (32-36)	38 (37-41)	<0.001
NYHA functional class III-IV	463 (50.5%)	549 (31.7%)	<0.001
Supported residential facility	57 (6.2%)	40 (2.3%)	<0.001
Diabetes mellitus	291 (31.7%)	493 (28.3%)	0.081
Insulin dependent diabetes mellitus	82 (8.9%)	96 (5.5%)	0.001
Atrial fibrillation	322 (35.0%)	543 (31.3%)	0.056
Stroke	107 (11.7%)	129 (7.4%)	<0.001
Peripheral vascular disease	156 (17.0%)	213 (12.3%)	0.001
Chronic kidney disease	429 (46.6%)	663 (38.1%)	<0.001
Hemoglobin (g/L)	121 (110-133)	130 (121-140)	<0.001
Anemia	412 (44.8%)	424 (24.4%)	<0.001
Coronary artery disease	691 (75.1%)	1,321 (76.0%)	0.659
Coronary artery bypass graft	115 (12.5%)	231 (13.3%)	0.624
Surgical aortic valve replacement	57 (6.2%)	89 (5.1%)	0.284
Left ventricular ejection fraction (%)	59 (50-63)	60 (55-65)	<0.001
LVEF <40%	92 (10.0%)	106 (6.1%)	<0.001
Mean pressure gradient (mm Hg)	42 (36-50)	42 (37-50)	0.453
Aortic valve area (cm^2^)	0.8 (0.6-0.9)	0.8 (0.7-0.9)	<0.001
Aortic peak velocity (m/s)	4.18 (3.8-4.5)	4.2 (3.85-4.5)	0.461
Prior BAV	553 (60.4%)	984 (56.6%)	0.064
Valve-in-valve TAVR	68 (7.4%)	94 (5.4%)	0.053
Nonfemoral access	16 (1.7%)	24 (1.4%)	0.576
Self-expanding valve	525 (57.6%)	1,003 (57.8%)	0.937
Procedure time (minutes)	63 (52-80)	60 (50-75)	0.001

BAV = Balloon Aortic Valvuloplasty (not bicuspid aortic valve); LVEF = left ventricular ejection fraction; STS = Society of Thoracic Surgeons; TAVR = transcatheter aortic valve replacement.

### Frailty and post-TAVR outcomes

Short-term outcomes are presented in Table 2. In-hospital (1.4% vs 0.5%; *P* = 0.016) and 30-day (2% vs 1%; *P* = 0.001) mortality was higher among the frail cohort. Frail patients were also significantly more likely to require admission to the intensive care unit (5% vs 2%; *P* < 0.001) and experience new nonhome discharge (14% vs 7%; *P* < 0.001). There was no difference in rates of other in-hospital complications including acute kidney injury, pacemaker implantation, vascular complication, and stroke.

**Table 2:** In-Hospital Outcomes Stratified by Frailty Status

	Frail	Not Frail	*P* Value
Intensive care unit admission	49 (5.3%)	39 (2.3%)	<0.001
Hospital stay (days)	4 (3-7)	4 (3-5)	<0.001
Nonhome discharge	125 (14.1%)	112 (6.6%)	<0.001
Stroke	16 (1.7%)	17 (1.0%)	0.132
Acute kidney injury	54 (5.9%)	78 (4.5%)	0.142
Pacemaker insertion	92 (10%)	167 (9.6%)	0.788
Vascular complication	24 (2.6%)	45 (2.6%)	1.000
Minor bleeding	15 (1.6%)	41 (2.4%)	0.273
Major bleeding	5 (0.5%)	7 (0.4%)	0.831
Mean pressure gradient (mm Hg)	8 (6-12)	9 (6-12)	0.395
Aortic valve area (cm^2^)	1.8 (1.4-2.1)	1.8 (1.5-2.2)	0.087
Death	13 (1.4%)	8 (0.5%)	0.016

Kaplan-Meier analysis showed 12-month all-cause mortality increased in stepwise fashion with increasing frailty (no frailty 4% vs BF or CF 7% vs BF + CF 16%; log-rank *P* < 0.001) (Supplemental Figure 1). Pairwise comparisons with Bonferroni correction confirmed statistically significant differences across all group pairs (no frailty vs BF or CF, *P* = 0.010; No Frailty vs BF + CF, *P* < 0.001; BF or CF vs BF + CF, *P* = 0.002). Separate Kaplan-Meier analyses demonstrated that both albumin and Rockwood CFS, when considered individually as markers of frailty, discriminated 12-month mortality: patients with albumin <35 g/L had higher mortality than those with albumin ≥35 g/L (10% vs 5%; log-rank *P* < 0.001) and patients with CFS ≥5 had higher mortality than those with CFS <5 (10% vs 5%, log-rank *P* < 0.001) (Supplemental Figure 2).

Cox proportional hazards regression adjusted for frailty, age, STS score, baseline atrial fibrillation, peripheral vascular disease, anemia, and chronic kidney disease. The model included 2,648 patients with 108 deaths and demonstrated good discriminative ability (concordance index 0.71; SE 0.03). With no frailty used as the reference group, BF or CF (adjusted HR [aHR]: 1.6; 95% CI: 1.0-2.4; *P* = 0.033) and BF + CF (aHR: 3.3; 95% CI: 1.9-5.6; *P* < 0.001) were independent predictors of 12-month all-cause mortality. Baseline atrial fibrillation (aHR: 1.6; 95% CI: 1.1-2.4; *P* = 0.013) and anemia (aHR: 1.9; 95% CI: 1.3-2.9; *P* = 0.001) also predicted this endpoint (Supplemental Table 2). Adjusted mortality curves derived from the multivariable Cox model demonstrated a persistent stepwise increase in mortality with increasing frailty after adjustment for baseline covariates (Figure 1).

**Figure 1:**
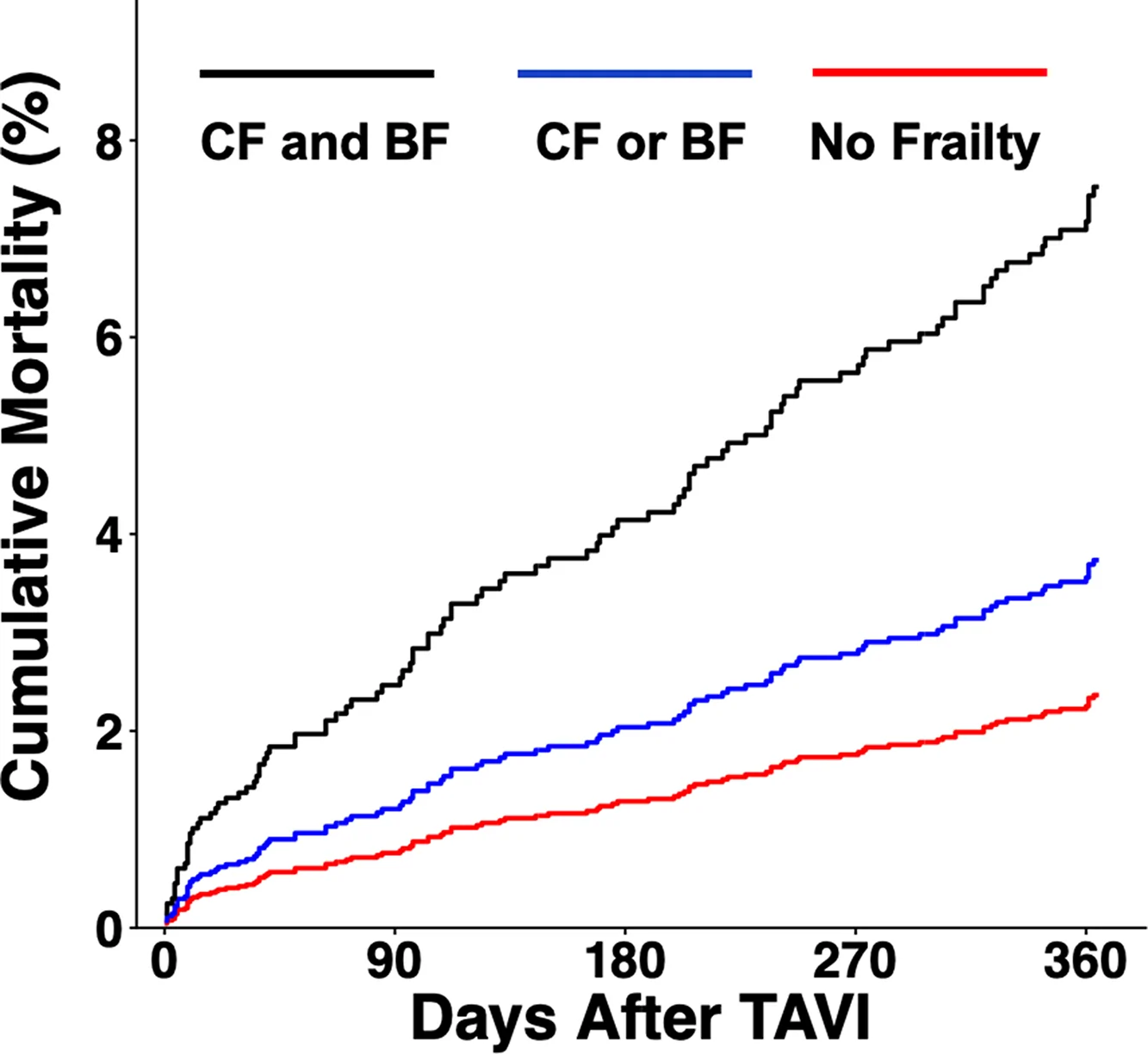
Adjusted 12-Month Mortality After Transcatheter Aortic Valve Replacement, Stratified by Frailty Status This figure displays the adjusted mortality curves derived from Cox proportional hazards models with adjustments for age, Society of Thoracic Surgeons Predicted Risk of Mortality Score, atrial fibrillation, chronic kidney disease, peripheral vascular disease, and anemia. To generate adjusted curves continuous covariates were fixed at their mean values, and categorical covariates were set to their reference category. The analysis demonstrates the stepwise increase in mortality with increasing frailty. BF = biochemical frailty; CF = clinical frailty; TAVR = transcatheter aortic valve replacement.

### Sensitivity analysis

Several prespecified sensitivity analyses were performed to evaluate the robustness of the primary findings. Firstly, to examine if frailty predicted poorer outcomes beyond the sickest and most unstable patients, we excluded urgent procedures for acute valve syndromes. A total of 2,461 patients were included with 96 deaths (Supplemental Table 3). Secondly, to assess whether the association between frailty and outcomes persisted in a lower-risk, less comorbid population, we restricted the cohort to contemporary procedures performed from 2019 onward. Overall, 2,371 patients were included with 93 deaths (Supplemental Table 4). Thirdly, to determine whether the prognostic impact of frailty extended beyond the index hospitalization, we conducted a landmark analysis excluding in-hospital deaths. A total of 2,630 patients were included with 87 deaths (Supplemental Table 5). In multivariable Cox regression, BF and CF remained an independent predictor of 12-month mortality in all sensitivity models.

### Addition of frailty to STS score

The STS score alone predicted 12-month mortality with an AUC of 0.608 (95% CI: 0.56-0.66). Incorporating frailty into the model improved the predictive performance to an AUC of 0.664 (95% CI: 0.62-0.71), representing an absolute increase of 0.056, which was statistically significant (DeLong test; *P* = 0.021) (Figure 2). To further quantify the relative contribution of each variable to model discrimination, a Shapley value decomposition was performed. Frailty contributed 0.085 to the total improvement in AUC above chance (0.5), whereas the STS score contributed 0.079. The total AUC gain above 0.5 was 0.164, with frailty accounting for 52% of this improvement. Addition of frailty also resulted in modest improvement incensoring adjusted continuous net reclassification (NRI 0.198; 95% CI: 0.095-0.297; *P* < 0.001). An unadjusted directional assessment of individual predicted risks showed that 57% of patients who died within 12 months were reclassified toward higher predicted mortality risk, whereas 66% of patients who remained event-free were reclassified toward lower predicted risk.

**Figure 2:**
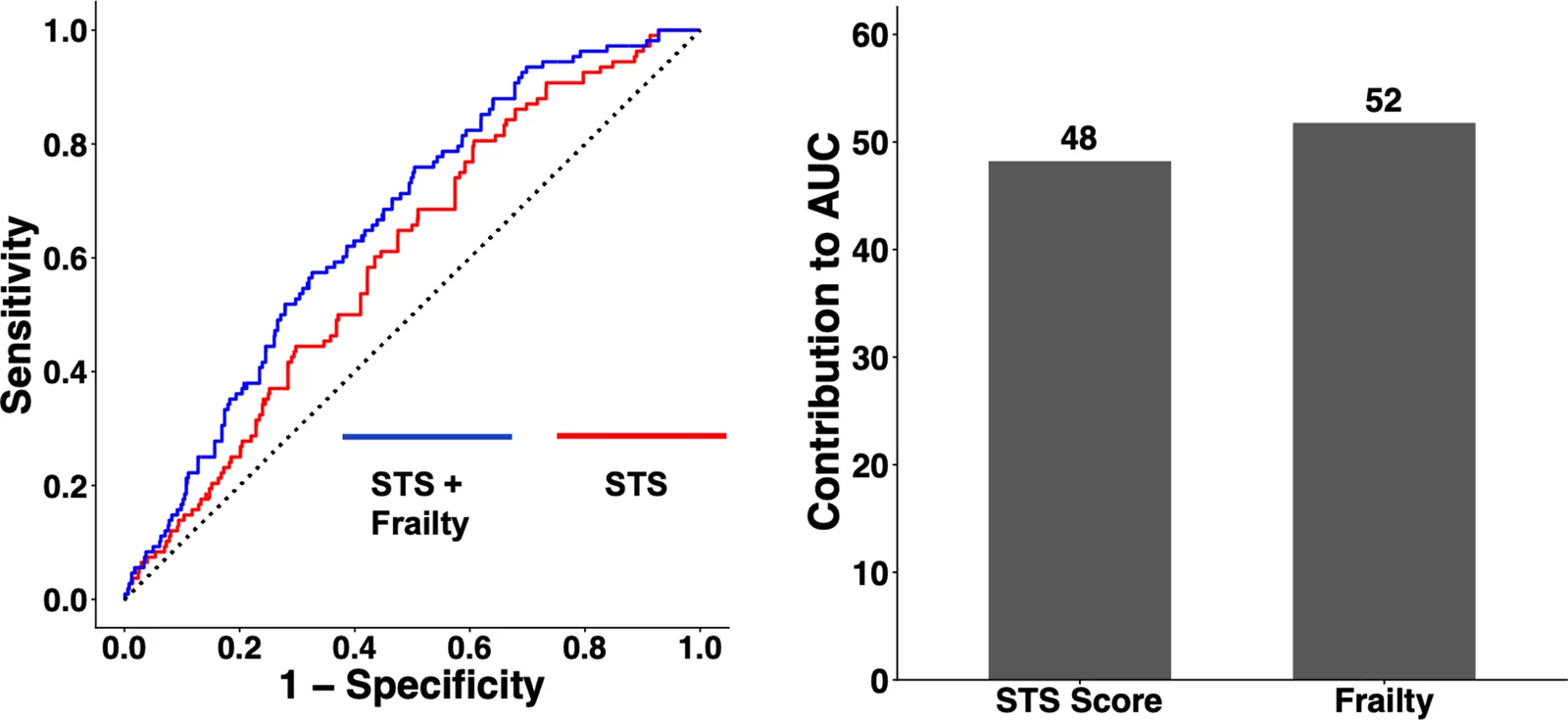
Addition of Frailty to the Society of Thoracic Surgeons Score for 12 Month Mortality Prediction The left panel shows the receiver operating characteristic curves for prediction of 12-month mortality. The red line represents the discrimination of the STS Score alone. The blue line represents the discrimination of STS Score combined with frailty. Addition of frailty resulted in a modest improvement in outcome prediction. The right panel shows Shapley value decomposition illustrating the proportion of the model's incrimental predictive contribution attributable to each predictor. AUC = area under the receiver-operating characteristic curve; STS = Society of Thoracic Surgeons.

**Central Illustration:**
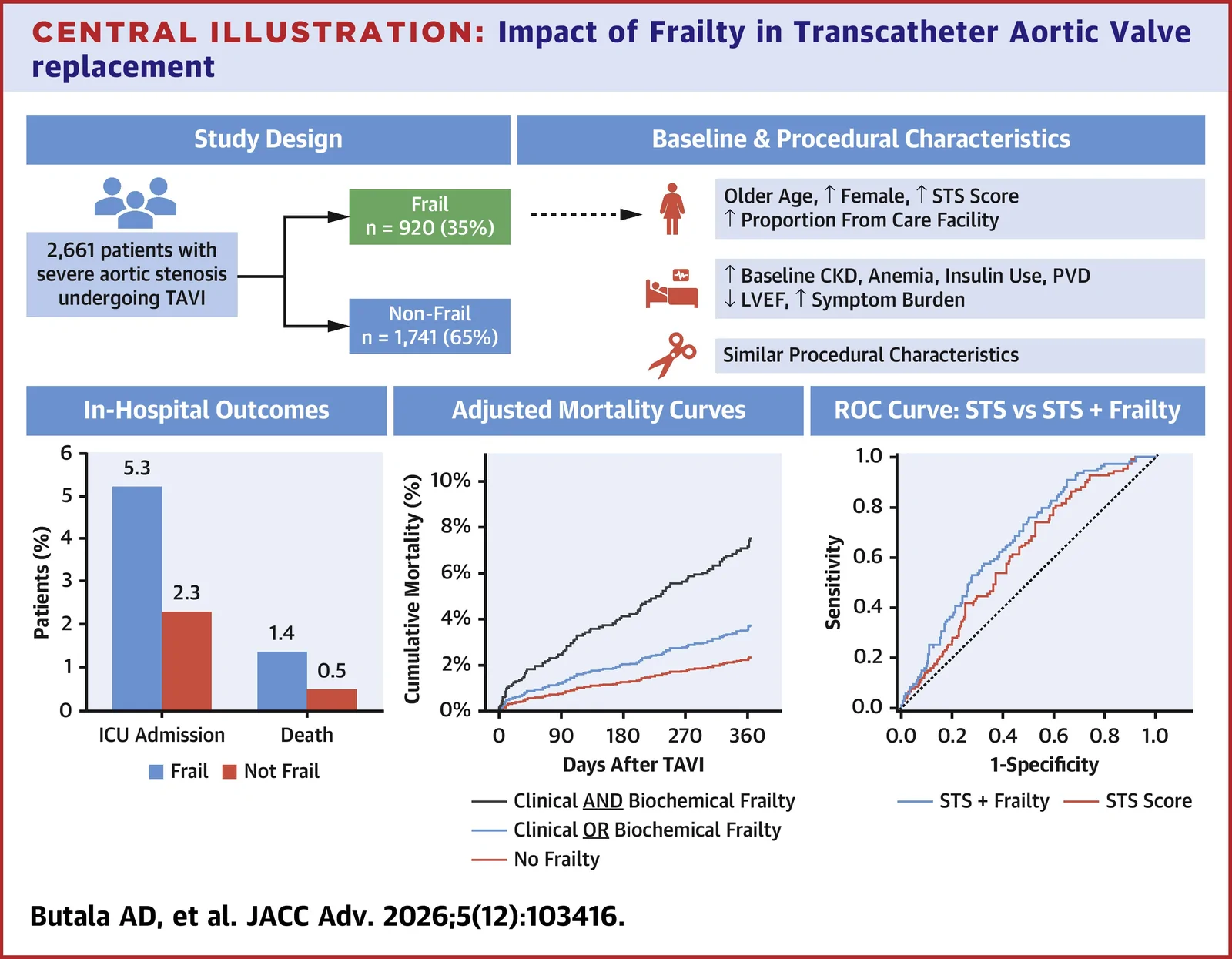
Impact of Frailty in Transcatheter Aortic Valve replacement Patients undergoing TAVR with frailty were older, more frequently resided in a supportive care facility, and had higher rates of cardiovascular comorbidities. Procedural characteristics were similar between groups. The bottom left panel demonstrates the higher rates of in-hospital adverse outcomes in frail patients. The bottom middle panel shows 12-month mortality remained significantly higher in the frail cohort after multivariable adjustment. The bottom right panel highlights modest improvement in mortality prediction when adding frailty to the STS score. CKD = chronic kidney disease; ICU = intensive care unit; LVEF = left ventricular ejection fraction; PVD = peripheral vascular disease; ROC = receiver-operating characteristic curve; STS = Society of Thoracic Surgeons; TAVR = transcatheter aortic valve replacement.

## Discussion

The primary findings from our large, multicenter study are: 1) frailty remains highly prevalent even in a contemporary, lower-risk TAVR cohort; 2) frailty is an independent predictor of increased 12-month mortality with the combination of biochemical and clinical frailty representing the highest-risk phenotype; and 3) frailty modestly improves TAVR mortality prediction when added to STS score.

More than one-in-three patients in our study were frail, underscoring the substantial burden of frailty among the TAVR population. Although this figure is consistent with previous literature,^19^, ^20^, ^21^ reported rates of frailty differ significantly. This is likely secondary to the lack of standardization in frailty assessment indices and the inclusion of patients with varying risk profiles. Importantly, there is no universally accepted biomarker for frailty. Although albumin is widely available and cost-effective, it should be viewed as a surrogate of biological vulnerability rather than a definitive biomarker of frailty. Interestingly, a contemporary analysis of the SCOPE I randomized trial by Tomii et al^22^ which shared a comparably low-risk cohort to our study, reported only 16.5% of patients as frail. The disparity may be explained by volunteer bias, whereby patients selected for clinical trials are likely healthier and less frail at baseline compared to our real-world all-comers population. Notably, we report significant rates of frailty in patients who may conventionally be considered robust: age <70 years (33% frail), STS scores <4 (30% frail), and severe obesity with body mass index ≥35 kg/m^2^ (40% frail). These findings support research by Mohiaddin et al^23^ who found more than 20% of patients aged between 55 to 74 with acute myocardial infarctions in the England and Wales were at least moderately frail. Similarly, Pinheiro et al have shown that increasing epicardial adipose tissue is associated with rising frailty in subjects with severe AS.^24^ Our study supplements the emerging literature highlighting the importance for clinicians to avoid the perception that frailty is isolated to older, underweight, and heavily comorbid groups. Adopting an individualized patient-centered approach with dedicated frailty assessment tools during the TAVR work up process is valuable.

Interestingly, our frail cohort had several clinical and echocardiographic markers of more advanced AS including a smaller aortic valve area, lower left ventricular ejection fraction and higher rates of NYHA functional class III-IV symptoms. In those patients with data available, the frail group also had significantly longer waiting times for TAVR. This raises an important consideration of whether the AS itself is contributing to frailty, whereby worsening valvulopathy and its physiological and hemodynamic implications further compromise already vulnerable patients. There are several mechanisms by which AS may exacerbate frailty: 1) worsening cardiac cachexia; 2) reducing exercise capacity leading to skeletal muscle atrophy; 3) impairing mitochondrial function in skeletal muscle due to chronic hypoperfusion; and 4) chronic inflammation secondary to increased circulating cytokine burden. With growing support for earlier application of valve intervention including in asymptomatic patients,^25^ further studies evaluating intervention in patients exhibiting initial features of frailty may be warranted.

This study also found an association between frailty and poor early postprocedural outcomes, in keeping with previous literature.^26^ The harmful effects of preprocedural frailty extended beyond the hospital admission, with frailty as an independent predictor of 12-month all-cause mortality. Building on existing literature regarding TAVR mortality prediction, this study highlights the additive value of incorporating both biochemical and clinical frailty assessments. In multivariable analysis, individuals who met both the criteria had more than double the risk of 12-month mortality compared to those meeting only a single criterion (aHR: 3.3 vs 1.6). Surprisingly, the correlation between increasing Rockwood CFS and lower albumin levels was weak (Spearman ρ = −0.19; *P* < 0.001). With frailty considered a multisystem syndrome, the weak correlation coefficient suggests albumin and the CFS are likely assessing distinct yet complementary domains within a broader phenotype. Our findings support the multicenter Frailty-AVR study, in which the essential frailty toolset, incorporating chair raises, cognitive impairment, hemoglobin, and albumin outperformed purely clinical indices such as the Short Physical Performance Battery and Fried criteria.^11^ This phenomenon was also replicated by Mizuno et al^27^ in their post hoc analysis of the POPEYE-AHF registry focused on patients with acute heart failure. Taken together, a growing body of evidence appears to suggest that frailty assessment should integrate both clinical and biochemical measures, as each capture distinct yet complementary frailty domains.

Our findings also demonstrate the additive value of incorporating frailty to the STS score during preprocedural assessment, with modest improvement in AUC (AUC STS 0.608 vs AUC STS + frailty 0.664; DeLong *P* = 0.021). Shapley value decomposition demonstrated that frailty contributed a similar proportion of predictive performance to the STS score. Importantly, the signal of modest predictive benefit remained consistent across censoring adjusted continuous NRI analysis. This is also in keeping with the analysis of the Japanese multicenter OCEAN registry (STS AUC: 0.61 vs STS + Frailty AUC: 0.64)^19^ by Shimura et al and single-center study of Swiss patients (STS C-statistic 0.64 vs STS + frailty C-statistic 0.68) by Schoenenberger et al.^28^ Although our improvements in AUC and NRI were both statistically significant, the absolute increase was modest and directional reclassification among patients who subsequently died was incremental. The extent to which this may meaningfully alter treatment decisions beyond current heart team assessment requires further evaluation. Our observational analysis does not support the use of frailty as an independent determinant of procedural eligibility and should not be used to establish treatment futility. Rather, frailty may serve as a useful adjunct in the preprocedural process: enhancing clinical assessment and individualized risk stratification, improving shared decision-making and patient counseling, and setting realistic expectations regarding post-TAVR outcomes.

Our findings highlight the need for future research to examine the modifiability of frailty and the resultant effects on postprocedural outcomes. Nutritional counseling, physical rehabilitation, and management of comorbidities may represent potentially promising pathways of preprocedural optimization. The benefit of these measures remains equivocal and there is a paucity of prospective evidence assessing frailty modification in TAVR. Upcoming clinical trials including FRAIL-AS Response^29^ will serve as important additions to the current literature.

### Study Limitations

There are important limitations in the present study. Firstly, BF was assessed using a single marker. Albumin is a nonspecific measure influenced by several biological processes including malnutrition, systemic inflammation, hepatic and renal dysfunction, and chronic illness. Furthermore, although often inherently linked, albumin may also reflect advanced comorbidity rather than frailty. In this context, it should be viewed as a surrogate of biological vulnerability and not a definitive marker of frailty. Analysis of other inflammatory, nutritional, and metabolic markers that may correlate with frailty would have provided a more comprehensive perspective but were not available. Secondly, the study evaluates mortality without assessing patient-centered outcomes. Current treatment goals for AS extend beyond survival with guidelines emphasizing improvement in symptoms, functional status, independence, and quality of life as major indications for valve intervention. Many frail patients may experience substantial symptomatic benefit despite having higher mortality risk. Consequently, mortality alone does not adequately define procedural success. Data regarding these patient focused outcomes would have provided a more holistic perspective. Thirdly, the large number of univariable comparisons performed for baseline characteristics introduces the potential for type I error, and statistically significant differences should therefore be interpreted with caution. Fourthly, our study represents an Australian TAVR cohort of predominantly Caucasian ethnicity. Comprising of a contemporary and lower-risk profile, the study is likely generalizable to many contemporary TAVR populations. However, differences in patient demographics, health care systems and referral practices should be considered when applying our findings.

## Conclusions

As TAVR continues to expand its therapeutic reach across lower risk populations, screening for frailty remains important. Clinical and biochemical frailty indices should be combined to optimize their predictive value. Consideration of frailty during preprocedural assessment can serve as a helpful adjunct to improve risk stratification and patient focused decision making. Development of preprocedural strategies to address frailty is critical.

Perspectives **COMPETENCY IN MEDICAL KNOWLEDGE 1:** Frailty is increasingly recognized as an important predictor of adverse outcomes following TAVR reflecting reduced physiological reserve beyond traditional surgical risk scores. This large, contemporary Australian study demonstrates biochemical and clinical frailty independently predicted 12-month mortality. Biochemical and clinical frailty are likely separate but overlapping phenotypes, and patients with both are a subgroup at particularly high risk of adverse outcomes. Incorporation of frailty into the STS risk model modestly improved discrimination for mortality prediction

**TRANSLATIONAL OUTLOOK:** Future work should focus on prospective validation of combined biochemical and clinical frailty models across broader TAVR populations and health care systems. Interventional studies are needed to determine whether targeted preprocedural frailty modification strategies can improve post-TAVR outcomes and risk prediction tools

## Funding support and author disclosures

Prof Stub is on the medical advisory boards of Edwards, Medtronic and Anteris; and his research is supported by grants from the National Health and Medical Research Council (NHMRC) of Australia and National Heart Foundation of Australia. Prof Walton is on the medical advisory boards of Medtronic, Abbott and Edwards. All other authors have reported that they have no relationships relevant to the contents of this paper to disclose.

## Supplementary material

Supplementary materia
