# Supplementary material for: The Impact of Clinical and Biochemical Frailty on Outcomes following Transcatheter Aortic Valve Replacement
Source: JACC Adv. 2026 Nov 3;5(12):103416. doi: 10.1016/j.jacadv.2026.103416 (PMC13755945; doi:10.1016/j.jacadv.2026.103416)
Supplement: Supplementary file 1 [file mmc1.docx]

**Supplementary Figure 1**

Description: 12-Month Cumulative Mortality Kaplan-Meier Curves Stratified by Frailty Status

**Supplementary Figure 2**

Description: 12-Month Cumulative Mortality Kaplan-Meier Curves Stratified by Albumin (left-panel) and Clinical Frailty Score (right-panel)

**Supplementary Table 1**

Description: Proportion of Missing Observations Among Variables Included Within Cox Regression Model

| **Variable** | **Number Missing** | **Percentage Missing** |
| --- | --- | --- |
| **Frailty** | 0 | 0% |
| **STS Score** | 0 | 0% |
| **Age** | 0 | 0% |
| **Prior AF** | 4 | 0.15% |
| **Prior CKD** | 0 | 0% |
| **Prior PVD** | 9 | 0.34% |
| **Anaemia** | 0 | 0% |

**Supplementary Table 2**

Description: Cox Hazard Regression Analysis 12-Month All-Cause Mortality

| **Variable** | **Unadjusted HR (95% CI)** | **Univariable p-value** | **Adjusted HR (95% CI)** | **Multivariable p-value** |
| --- | --- | --- | --- | --- |
| **Biochemical or Clinical Frailty** | 1.87 (1.23–2.85) | 0.004 | 1.59 (1.04–2.44) | 0.033 |
| **Biochemical and Clinical Frailty** | 4.59 (2.75–7.68) | <0.001 | 3.27 (1.91–5.59) | <0.001 |
| **Age (per year)** | 1.03 (1.00–1.06) | 0.048 | 1.01 (0.98–1.04) | 0.522 |
| **Male** | 1.07 (0.73–1.58) | 0.720 | — | — |
| **STS Score (per unit)** | 1.07 (1.02–1.12) | 0.003 | 1.01 (0.96–1.07) | 0.576 |
| **Public Hospital** | 1.05 (0.71–1.55) | 0.805 | — | — |
| **Coronary Artery Disease** | 1.11 (0.71–1.73) | 0.661 | — | — |
| **Atrial Fibrillation** | 1.83 (1.26–2.68) | 0.002 | 1.64 (1.11–2.41) | 0.013 |
| **Stroke** | 1.25 (0.68–2.27) | 0.471 | — | — |
| **Chronic Kidney Disease** | 1.53 (1.05–2.23) | 0.028 | 1.11 (0.75–1.65) | 0.602 |
| **Diabetes** | 1.07 (0.71–1.62) | 0.741 | — | — |
| **Peripheral Vascular Disease** | 1.86 (1.18–2.93) | 0.007 | 1.55 (0.98–2.46) | 0.063 |
| **Anaemia** | 2.53 (1.73–3.69) | <0.001 | 1.92 (1.29–2.86) | 0.001 |
| **Left Ventricular Ejection Fraction<40%** | 1.60 (0.88–2.92) | 0.125 | — | — |
| **Valve-in-Valve** | 1.02 (0.45–2.33) | 0.956 | — | — |

**Supplementary Table 3**

Description: Sensitivity Analysis 1: Cox Hazard Regression for 12-Month All-Cause Mortality Excluding Urgent Inpatient TAVI for Acute Valve Syndrome

| **Variable** | **Unadjusted HR (95% CI)** | **Univariable p-value** | **Adjusted HR (95% CI)** | **Multivariable p-value** |
| --- | --- | --- | --- | --- |
| **Biochemical or Clinical Frailty** | 1.87 (1.23–2.85) | 0.0076 | 1.53 (0.98–2.40) | 0.0633 |
| **Biochemical and Clinical Frailty** | 4.77 (2.72–8.34) | <0.0001 | 3.39 (1.91–6.04) | <0.0001 |
| **Age** | 1.05 (1.01–1.08) | 0.0060 | 1.03 (0.99–1.06) | 0.110 |
| **Male** | 1.15 (0.76–1.74) | 0.514 | — | — |
| **STS Score** | 1.07 (1.03–1.12) | 0.0022 | 1.01 (0.96–1.07) | 0.592 |
| **Public Hospital** | 0.98 (0.65–1.50) | 0.940 | — | — |
| **Coronary Artery Disease** | 1.18 (0.73–1.91) | 0.511 | — | — |
| **Atrial Fibrillation** | 1.75 (1.17–2.61) | 0.0065 | 1.51 (1.01–2.27) | 0.0461 |
| **Stroke** | 1.42 (0.78–2.61) | 0.252 | — | — |
| **Chronic Kidney Disease** | 1.52 (1.02–2.27) | 0.0393 | 1.12 (0.74–1.69) | 0.605 |
| **Diabetes** | 1.19 (0.77–1.83) | 0.438 | — | — |
| **Peripheral Vascular Disease** | 2.04 (1.28–3.26) | 0.0029 | 1.72 (1.07–2.78) | 0.0264 |
| **Anaemia** | 2.47 (1.65–3.68) | <0.0001 | 1.86 (1.22–2.82) | 0.0037 |
| **Left Ventricular Ejection Fraction<40%** | 1.77 (0.92–3.40) | 0.0878 | 1.45 (0.75–2.83) | 0.273 |
| **Valve-in-valve** | 0.67 (0.21–2.10) | 0.487 | — | — |

**Supplementary Table 4**

Description: Sensitivity Analysis 2: Cox Hazard Regression for 12-Month All-Cause Mortality Excluding TAVI Performed Before 2019

| **Variable** | **Unadjusted HR (95% CI)** | **Univariable p-value** | **Adjusted HR (95% CI)** | **Multivariable p-value** |
| --- | --- | --- | --- | --- |
| **Biochemical or Clinical Frailty** | 1.75 (1.11–2.77) | 0.0165 | 1.42 (0.89–2.27) | 0.138 |
| **Biochemical and Clinical Frailty** | 4.58 (2.64–7.94) | <0.0001 | 2.99 (1.67–5.36) | <0.001 |
| **Age** | 1.03 (1.00–1.06) | 0.0470 | 1.01 (0.98–1.04) | 0.424 |
| **Male** | 1.23 (0.80–1.89) | 0.341 | — | — |
| **STS Score** | 1.07 (1.02–1.12) | 0.0035 | 1.01 (0.96–1.07) | 0.716 |
| **Public Hospital** | 1.11 (0.71–1.74) | 0.654 | — | — |
| **Coronary Artery Disease** | 1.19 (0.71–1.99) | 0.513 | — | — |
| **Atrial Fibrillation** | 1.78 (1.18–2.67) | 0.0058 | 1.59 (1.05–2.40) | 0.0299 |
| **Stroke** | 1.17 (0.59–2.32) | 0.661 | — | — |
| **Chronic Kidney Disease** | 1.56 (1.04–2.35) | 0.0317 | 1.10 (0.72–1.68) | 0.663 |
| **Diabetes** | 0.99 (0.63–1.56) | 0.971 | — | — |
| **Peripheral Vascular Disease** | 1.87 (1.15–3.04) | 0.0117 | 1.53 (0.93–2.51) | 0.0926 |
| **Anaemia** | 2.72 (1.81–4.09) | <0.0001 | 2.06 (1.33–3.19) | 0.0011 |
| **Left Ventricular Ejection Fraction <40%** | 1.91 (1.04–3.50) | 0.0370 | 1.57 (0.84–2.94) | 0.154 |
| **Valve-in-valve** | 1.09 (0.48–2.50) | 0.832 | — | — |

**Supplementary Table 5**

Description: Landmark Analysis: Cox Hazard Regression for 12-Month All-Cause Mortality Excluding In-hospital Mortality

| **Variable** | **Unadjusted HR (95% CI)** | **Univariable p-value** | **Adjusted HR (95% CI)** | **Multivariable p-value** |
| --- | --- | --- | --- | --- |
| **Biochemical or Clinical Frailty** | 2.01 (1.27–3.18) | 0.0029 | 1.69 (1.06–2.69) | 0.0265 |
| **Biochemical and Clinical Frailty** | 3.86 (2.09–7.12) | <0.0001 | 2.72 (1.46–5.08) | 0.0017 |
| **Age** | 1.04 (1.01–1.08) | 0.0177 | 1.02 (0.99–1.05) | 0.246 |
| **Male** | 1.31 (0.84–2.05) | 0.235 | — | — |
| **STS Score** | 1.04 (0.98–1.10) | 0.171 | — | — |
| **Public Hospital** | 1.15 (0.75–1.77) | 0.524 | — | — |
| **Coronary Artery Disease** | 0.99 (0.61–1.61) | 0.970 | — | — |
| **Atrial Fibrillation** | 2.10 (1.38–3.19) | 0.0006 | 1.88 (1.23–2.87) | 0.0037 |
| **Stroke** | 1.43 (0.76–2.70) | 0.264 | — | — |
| **Chronic Kidney Disease** | 1.60 (1.05–2.43) | 0.0290 | 1.17 (0.76–1.80) | 0.480 |
| **Diabetes** | 1.10 (0.70–1.74) | 0.679 | — | — |
| **Peripheral Vascular Disease** | 1.98 (1.20–3.26) | 0.0075 | 1.71 (1.03–2.83) | 0.0370 |
| **Anaemia** | 2.69 (1.77–4.11) | <0.0001 | 2.04 (1.31–3.17) | 0.0017 |
| **Left Ventricular Ejection Fraction <40%** | 1.32 (0.64–2.74) | 0.450 | — | — |
| **Valve-in-valve** | 0.87 (0.32–2.38) | 0.789 | — | — |
